# YOLO-Tryppa: A Novel YOLO-Based Approach for Rapid and Accurate Detection of Small Trypanosoma Parasites

**DOI:** 10.3390/jimaging11040117

**Published:** 2025-04-15

**Authors:** Davide Antonio Mura, Luca Zedda, Andrea Loddo, Cecilia Di Ruberto

**Affiliations:** Department of Mathematics and Computer Science, University of Cagliari, Via Ospedale 72, 09124 Cagliari, Italy; cecilia.dir@unica.it

**Keywords:** computer vision, deep learning, image processing, Trypanosoma detection, YOLO-based architectures, lightweight detection models

## Abstract

Early detection of Trypanosoma parasites is critical for the prompt treatment of trypanosomiasis, a neglected tropical disease that poses severe health and socioeconomic challenges in affected regions. To address the limitations of traditional manual microscopy and prior automated methods, we propose YOLO-Tryppa, a novel YOLO-based framework specifically engineered for the rapid and accurate detection of small Trypanosoma parasites in microscopy images. YOLO-Tryppa incorporates ghost convolutions to reduce computational complexity while maintaining robust feature extraction and introduces a dedicated P2 prediction head to improve the localization of small objects. By eliminating the redundant P5 prediction head, the proposed approach achieves a significantly lower parameter count and reduced GFLOPs. Experimental results on the public Tryp dataset demonstrate that YOLO-Tryppa outperforms the previous state of the art by achieving an AP50 of 71.3%, thereby setting a new benchmark for both accuracy and efficiency. These improvements make YOLO-Tryppa particularly well-suited for deployment in resource-constrained settings, facilitating more rapid and reliable diagnostic practices.

## 1. Introduction

Neglected tropical diseases (NTDs) continue to impose severe health and socioeconomic burdens on vulnerable populations worldwide. Among these, trypanosomiasis caused by Trypanosoma parasites presents a significant diagnostic challenge [1], particularly in regions such as sub-Saharan Africa and Latin America. Conventional diagnosis via manual microscopy of blood smears is not only labor-intensive and subjective but also dependent on specialized expertise, often resulting in delays in diagnosis and treatment. This scenario underscores the urgent need for automated, reliable, and scalable diagnostic tools [2].

Recent advances in deep learning have transformed medical imaging and real-time object detection. Convolutional neural networks (CNNs) and, in particular, the YOLO (You Only Look Once) [3] family of models have demonstrated exceptional speed and accuracy in various detection tasks. Our previous work, YOLO-Para [4], adapted a custom YOLOv8 framework for malaria parasite detection, successfully capturing subtle morphological features in challenging microscopy images. Building on that success, we now extend our approach to the domain of trypanosome detection.

In this work, we introduce YOLO-Tryppa, a tailored detection framework explicitly engineered for the identification of Trypanosoma brucei. YOLO-Tryppa incorporates targeted architectural modifications designed to improve the localization of small parasites, which are prevalent in the Tryp dataset [5]. We used the YOLOv11m architecture and propose several key modifications, like the use of ghost convolutions instead of standard convolutional layers, to reduce computational complexity while maintaining accuracy. Additionally, on the one hand, we introduce a dedicated *P2* prediction head to specialize in detecting small objects, and, on the other hand, we remove the prediction head for larger objects, thereby aligning the architecture with the specific characteristics of trypanosome images. YOLO-Tryppa distinguishes itself from prior work [4] by emphasizing real-time detection capabilities while rigorously refining the YOLO framework for enhanced small object detection.

The development and evaluation of YOLO-Tryppa are supported by the Tryp dataset [5], a comprehensive collection of microscopy images with meticulously annotated bounding boxes for Trypanosoma brucei. This dataset captures diverse and complex presentations of the parasite, providing a robust benchmark for assessing detection performance. By tailoring the YOLO-Para architecture with these strategic modifications, our work demonstrates the adaptability of deep learning models in medical diagnostics. It contributes a valuable tool to enhance disease screening in resource-constrained environments.

The key contributions of this work include the following:

- A clear exposition of the challenges in diagnosing trypanosomiasis and the motivation for an automated solution;
- The development of YOLO-Tryppa, a novel detection framework with architectural innovations for enhanced small object detection;
- Extensive evaluation on the Tryp dataset, yielding significant improvements in detection performance and computational efficiency.

The remainder of this paper is organized as follows. In Section 2, we review the related work on Trypanosoma detection. Section 3 describes the materials and methods used in this study, including description of the dataset, the design and implementation of YOLO-Tryppa, and the architectural changes made to optimize small object detection. Then, Section 4.3, presents the experimental evaluation and the obtained results. Finally, in Section 5 and Section 6, we discuss the current limitations of our detection approach and outline future research directions.

## 2. Related Work

This section reviews the literature on object detection in medical imaging, focusing on parasite detection in microscopy images, establishing the context for our proposed YOLO-Tryppa framework, designed to enhance the detection of Trypanosoma brucei brucei in resource-constrained settings. Specifically, this section is organized into four subsections: Section 2.1 addresses the historical progression of object detection methods in medical imaging, including the transition to deep learning, Section 2.2 presents the unique challenges and methodologies associated with parasite detection in microscopy images, Section 2.3 explores the role of attention mechanisms in enhancing detection accuracy by focusing on salient image features, and Section 2.4 examines lightweight object detection models suitable for resource-constrained environments.

### 2.1. Evolution of Object Detection in Medical Imaging

Traditional approaches to medical imaging have relied heavily on handcrafted features and classical computer vision techniques [6]. While these methods offered early insights, their performance was often hindered by sensitivity to noise and variability in clinical data. The advent of CNNs [7] marked a paradigm shift by enabling robust, hierarchical feature extraction directly from raw images. This breakthrough originated with the development of R-CNN [8], Fast R-CNN [9], and single-shot detectors, which collectively improved both detection accuracy and processing speed [10]. Modern deep learning architectures have redefined object detection by striking an effective balance between high accuracy and real-time performance. Models such as the YOLO family [3], Faster R-CNN [9], and RetinaNet [11] employ end-to-end training pipelines that predict bounding boxes and class probabilities directly from full images [4,12]. These models have been successfully adapted to medical imaging contexts by fine tuning them to capture subtle pathological features despite challenges like low contrast and variable morphology [13]. Custom modifications and transfer learning strategies have further bolstered their applicability to specialized tasks, including the detection of parasitic infections.

### 2.2. Parasite Detection in Microscopy Images

Detecting parasites in microscopy images is inherently challenging due to the low contrast between parasites and surrounding tissues, high morphological variability, and the presence of imaging artifacts [14]. Trypanosomiasis, a significant public health concern, affects regions across South America, South Asia, Southeast Asia, and Sub-Saharan Africa [15]. The disease, transmitted by blood-sucking insects such as tsetse flies and tabanids, impacts both humans and animals, leading to serious zoonotic consequences. Although molecular techniques like polymerase chain reaction (PCR) and immunological assays remain the gold standard for detection, they demand skilled personnel, involve multiple processing steps, and require expensive equipment [16]. In contrast, microscopic examination offers a rapid and cost-effective diagnostic alternative. However, its low sensitivity and the variability in interpretation among technicians underscore the need for automated, computer-aided diagnostic (CAD) systems [17,18]. The integration of artificial intelligence in CAD systems holds promise for the standardization and acceleration of the detection process, particularly in resource-limited settings.

Current state-of-the-art research on parasite detection in microscopy images predominantly focuses on the identification of malaria parasites. For instance, in [19], three pre-trained models were employed alongside transfer learning techniques to accurately identify and classify malaria parasites. Similarly, in [20], a CNN integrated with a random forest algorithm was utilized to detect Plasmodium malaria parasites. With regard to Trypanosoma localization, random forest-based machine learning approaches were applied in [21] to extract features from microscopy images, facilitating the identification and quantification of the parasite. Additionally, Jung et al. [22] used the ResNet18 model on datasets derived from microscope video recordings of blood smears to detect the presence or absence of parasites. Furthermore, in [23], ResNet50 was utilized to identify Trypanosoma in images, and the trained model was further validated as an autonomous screening system using a vector database constructed with images processed through the K-Nearest Neighbor algorithm.

### 2.3. Integration of Attention Mechanisms

Attention mechanisms have become critical in enhancing CNN architectures for medical image analysis. These mechanisms enable models to focus selectively on the most relevant regions within an image, with techniques such as spatial, channel, and self-attention [24] significantly improving the accuracy of feature localization while mitigating the impact of background noise. This targeted approach reduces the rate of false positives and improves the detection of subtle and dispersed features [25,26]. Moreover, recent innovations in attention module design can facilitate dynamic feature weighting during the training and inference stages, thereby further optimizing detection performance. This capability is particularly essential for the accurate identification of parasites in complex microscopy images [27].

### 2.4. Lightweight Object Detectors

Deploying object detection models in resource-constrained environments, such as field clinics and remote laboratories, necessitates architectures that balance accuracy with computational efficiency [28,29,30]. Traditional deep learning-based object detectors, such as Faster R-CNN and RetinaNet, offer high detection accuracy but require substantial processing power, making them less suitable for real-time applications on edge devices [31,32]. To address this limitation, lightweight object detectors have been developed to provide real-time performance with reduced computational overhead [33].

Models like MobileNet-SSD [34] leverage depthwise separable convolutions and parameter-efficient architectures to maintain detection accuracy while significantly lowering inference time. The YOLO family, particularly custom YOLO architectures specialized for efficient resource utilization [35], has demonstrated promising results in mobile and embedded AI applications by reducing model complexity without drastically compromising performance. Similarly, EfficientDet [36] employs a compound scaling strategy to optimize network depth, width, and resolution, making it a viable choice for medical imaging tasks where both precision and efficiency are crucial.

## 3. Materials and Methods

In this section, we detail the materials and methods employed in the development and evaluation of YOLO-Tryppa. First, in Section 3.1, we describe the acquisition, preparation, and annotation of the Tryp dataset, which comprises microscopy images of unstained thick blood smears. Next, Section 3.2 outlines the experimental setup, including the design of the detection framework and training strategies, with a comparison of single-stage and two-stage object detectors. Section 4.1 presents the metrics and procedures used to evaluate the robustness and accuracy of YOLO-Tryppa, focusing on standard object detection measures such as AP, AP50, and the F1 score. Lastly, Section 3.3 provides a detailed explanation of ghost convolution, an efficient computational technique integrated into the model to reduce complexity without compromising performance.

### 3.1. Dataset

The Tryp dataset [5] is, to the best of our knowledge, the first, most comprehensive, and only publicly available collection of microscopy images of unstained thick blood smears specifically curated for the detection of Trypanosoma parasites. It comprises 3085 annotated images of infected (positive) blood samples and 93 images from non-infected (negative) blood samples. The images were acquired using two different microscopy setups, namely an IX83 inverted Olympus microscope and an Olympus CKX53 microscope, yielding varying resolutions: 1360×1024, 1920×1080, and 720×404 pixels.

Annotations, provided as tight bounding boxes around the parasite regions, were generated through a two-stage process using both Roboflow and Labelme, ensuring high consistency and quality. The dataset is partitioned into training, validation, and testing sets following a 60:20:20 ratio for the annotated images.

The distribution of parasite instances across the sets shows considerable variability. The training set contains 27,489 parasite instances, the validation set includes 8697 instances, and the test set comprises 9094 instances. When normalized by the number of images, these figures indicate a consistent ratio of images to parasite instances of ∼14.6 across the dataset.

**Figure 1:**
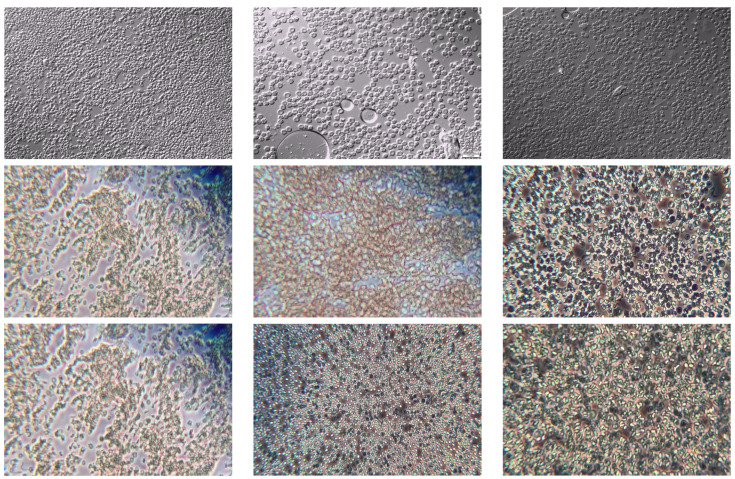
Representative samples from the Tryp dataset. The first row shows samples captured with the IX83 microscope, while the second and third rows display images obtained using the CKX53 microscope.

Figure 1 presents a representative selection of images from the dataset.

### 3.2. Object Detectors

Object detection methods in computer vision can be broadly categorized into single-stage and two-stage approaches, each offering distinct trade-offs between speed and accuracy. Single-stage detectors, such as YOLO [3] and SSD [34], perform object localization and classification in a single forward pass. This direct regression of bounding boxes and class probabilities from full images enables high processing speeds, making them suitable for real-time applications. However, this approach may sometimes compromise localization accuracy, particularly when detecting small or densely clustered objects. In contrast, two-stage detectors exemplified by architectures like Fast R-CNN [8] and Faster R-CNN [9] employ an initial stage to generate region proposals followed by a second stage that refines these proposals through classification and bounding-box regression. Although this two-step process generally incurs higher computational costs and longer inference times, it tends to yield superior localization precision. The selection between these methods ultimately depends on the application’s specific requirements, balancing the need for speed against the demand for accuracy.

### 3.3. Ghost Convolution

Ghost convolution is an innovative approach to reduce the computational cost of standard convolutional operations [37]. Traditional convolutional layers generate a large number of output feature maps by applying numerous convolution kernels, many of which can be redundant. The core idea behind ghost convolution is that a significant portion of these feature maps can be approximated through inexpensive linear operations rather than being computed directly via costly convolutions.

In practice, the process begins by applying a standard convolution to the input feature map to produce a compact set of intrinsic feature maps. These intrinsic maps capture the essential features of the input. Instead of directly computing all desired feature maps, ghost convolution synthesizes additional—or ghost—feature maps by applying a cheap linear transformation to the intrinsic features. This transformation is typically realized using operations such as depthwise convolutions.

Mathematically, if *X* represents the input feature maps and $W^{\prime }$ denotes the convolution kernels used to generate the intrinsic feature maps, then the intrinsic features ($Y^{\prime }$) are computed as in Equation (1):

(1) $$Y^{\prime }=X*W^{\prime }.$$

Subsequently, an inexpensive linear operation (*G*) is applied to $Y^{\prime }$ to generate the ghost features ($Y_{g}$) so that the final output (*Y*) can be approximated as follows:

(2) $$Y=Y^{\prime }+Y_{g}.$$

This strategy preserves the essential information captured by the intrinsic features and enriches the representation with additional details while significantly reducing the number of parameters and floating-point operations required. The result is a more efficient convolutional layer that maintains performance while offering substantial computational savings.

### 3.4. The Proposed Architecture: YOLO-Tryppa

**Figure 2:**
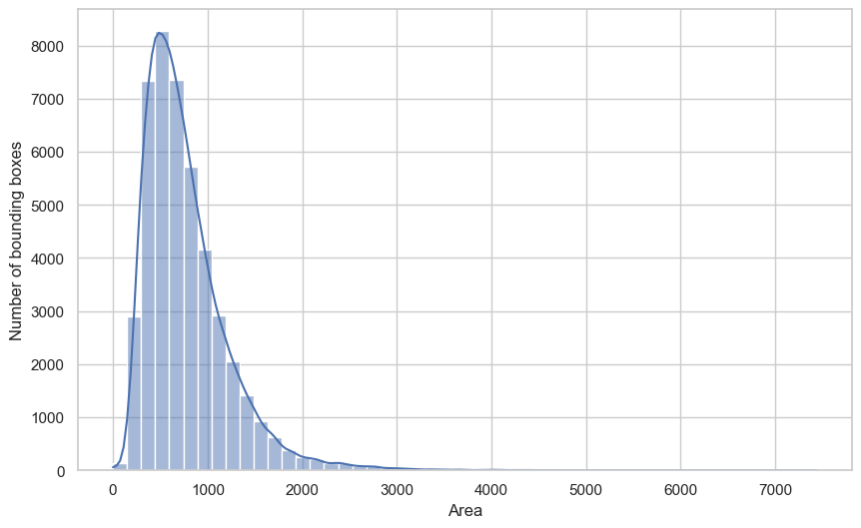
Parasite area distribution. The X-axis contains the area of the parasite bounding box, while the y-axis contains the frequencies of each area.

Our efforts in designing the YOLO-Tryppa architecture were directed toward two primary objectives: first, to enhance the localization of small parasites in blood smear images and, second, to develop models that are both lightweight and capable of real-time performance. To justify the first objective, in Figure 2, we present the area distribution following the COCO standard [38]. The distribution clearly reveals a bias toward small objects, with 78.4% of the objects classified as small having an area of less than $32^{2}$ pixels and the remaining 21.8% classified as medium-sized with areas between $32^{2}$ and $96^{2}$ pixels.

Building upon our previous work [4], detectors for small parasites require the use of the lower layers of the backbone in the chosen base architecture. However, doing so increases the number of parameters in the specialized architectures due to the presence of additional prediction heads and a higher number of GFLOPs. Therefore, to build our YOLO-Tryppa, we start from the YOLOv11m architecture, which offers a reliable trade-off between performance and real-time capabilities [39].

We then substitute all convolutional layers in the model architecture, including submodules, with ghost convolutions, except for the first two layers, similarly to the work of Cao et al. [35]. Next, we add a prediction head called *P2* and introduce a novel branch stemming from the *C2* feature map [3] that is specialized for the detection of small objects. Finally, we remove the *P5* prediction head, which is designed for larger objects that are absent from the considered dataset.

**Figure 3:**
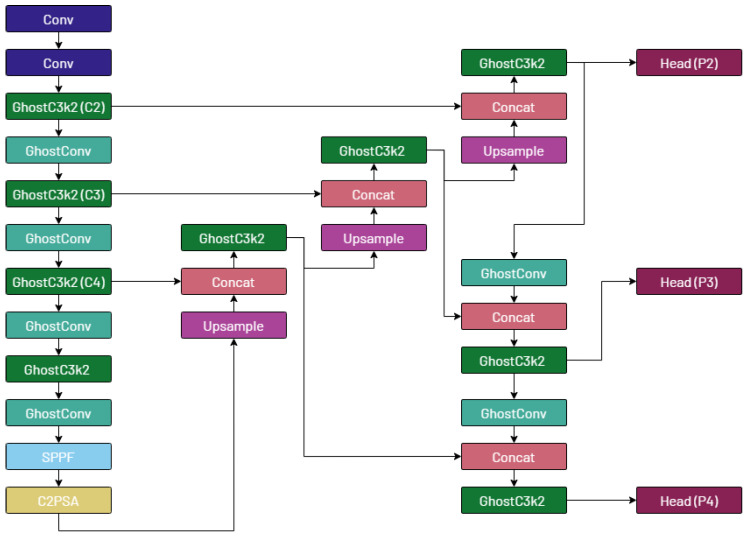
Visual representation of the YOLO-Tryppa architecture. On the left, the backbone is depicted, followed by the model’s neck, which leads to the three prediction heads: P2, P3, and P4.

The proposed YOLO-Tryppa architecture is visually represented in Figure 3.

## 4. Experimental Evaluation

This section presents the full range of experiments performed with the goal of achieving reliable and fast Trypanosoma detection. Specifically, in Section 4.1, we report the analyzed metrics and their clinical significance. In Section 4.2, we detail the experimental setup, including the hardware, training parameters, and the used optimization techniques. Section 4.3 provides a comprehensive evaluation of detection performance across different YOLO architectures, highlighting the comparative advantages of YOLO-Tryppa. Section 4.4 presents an ablation study to isolate and analyze the impact of individual architectural components on model performance. Finally, Section 4.5 presents qualitative results that visually demonstrate the effectiveness of YOLO-Tryppa in detecting Trypanosoma parasites under challenging conditions, complementing quantitative evaluations.

### 4.1. Detection Metrics

In evaluating detection performance, it is essential to quantify both the accuracy of object localization and the reliability of classification. Two standard metrics used in object detection are Average Precision (AP) and AP50. These metrics are built upon the concepts of True Positives (TPs), False Positives (FPs), and False Negatives (FNs). Here, TP represents correctly detected objects, FP corresponds to incorrect detections, and FN denotes objects that were missed by the detector. The decision on whether a predicted bounding box is a TP or an FP is based on the Intersection over Union (IoU) criterion, which measures the overlap between the predicted bounding box and the ground truth. An IoU threshold of 0.5 is typically employed, meaning that if the IoU exceeds 0.5, the detection is considered a match.

Average precision quantifies the area under the precision–recall curve. Precision (*P*) is defined as in Equation (3):

(3) $$P=\frac{TP}{TP+FP}\;.$$

Recall (*R*) is defined as in Equation (4):

(4) $$R=\frac{TP}{TP+FN}\;.$$

Different pairs of precision and recall values are obtained by varying the detection confidence threshold, which can be plotted to form the precision–recall curve. The AP is then computed as defined in Equation (5):

(5) $$AP=\int_{0}^{1}P\left(R\right)\;dR\;,$$

where P(R) is the precision as a function of recall. AP provides a comprehensive measure of detection performance, with higher values indicating a better balance between precision and recall.

AP50 is a specific case of AP where the IoU threshold is fixed at 0.5. This metric, denoted as AP50, evaluates detection performance under the condition of a predicted bounding box being considered a true positive if its IoU with a ground-truth box is at least 0.5. AP50 is widely used because it offers a straightforward evaluation that balances localization and classification accuracy, making it a common baseline in object detection benchmarks.

To balance the trade-off between precision and recall, we compute the F1 score (F1). As defined in Equation (6), it is the harmonic mean between P and R:

(6) $$F1=2\times \frac{P\times R}{P+R}$$

### 4.2. Experimental Setup

Our experiments were performed on a workstation featuring an RTX 4060 Ti GPU with 16 GB of VRAM and an Intel Core i5-13400 processor. Each model was trained for 40 epochs using a learning rate of $3\times 10^{-4}$, and the best-performing models were selected based on the highest AP score on the validation set. A batch size of 16 was used to train each model, except for the YOLO Para architecture. Due to memory constraints for the YOLO Para variants, a batch size of 1 was employed. The YOLO-Tryppa architecture was developed using the Ultralytics repository [3]. We also included the original YOLOv11 augmentation setup and kept the image size to match the COCO standard of 640×640. For optimization, we combined the AdamW optimizer with the original YOLOv11 loss, incorporating three components: distributed focal loss, bounding-box regression loss, and class probability loss. Its loss is defined in Equation (7):

(7) $$L_{\mathrm{YOLOv}11}=L_{\mathrm{cls}}+L_{\mathrm{box}}+L_{\mathrm{dfl}}\;,$$

where $L_{\mathrm{cls}}$ quantifies the difference between predicted and true class probabilities via cross-entropy, ensuring accurate categorization; $L_{\mathrm{box}}$ minimizes the error between predicted and actual bounding boxes using metrics like IoU; and $L_{\mathrm{dfl}}$ adjusts weights for challenging samples to improve detection accuracy. The model’s multi-stage design further refines feature extraction for superior object detection [40].

To address the challenges posed by class imbalance, we adopted a single-class approach where we exclusively predict the presence of parasites. This decision simplifies the learning task and allows the model to focus solely on parasite detection, avoiding potential biases stemming from under-represented non-parasitic classes.

### 4.3. Experimental Results

To ensure fairness and comparability across experiments, we first trained both the large and medium-sized YOLO architectures (versions 5, 8, and 11) using the same experimental setup as our proposed YOLO-Tryppa architecture. This strategy was adopted not only to maintain fairness but also to investigate whether increasing the number of parameters and, consequently, the complexity and size of the models can lead to enhanced detection performance. The full range of analyzed metrics and experiments is depicted in Table 1.

Our experiments reveal that the larger counterpart exhibits inferior performance for each off-the-shelf model. Notably, YOLOv8 displays the most significant variability, with the medium-sized model achieving an AP50 of 68.4%, compared to only 54.4% for the large model. Nonetheless, the baseline models outperform the other three architectures, RetinaNet, Faster R-CNN, and YOLOv7, by 5.4% in terms of AP50. It is important to note that both RetinaNet and Faster R-CNN used a larger image size of 1333×800 in the previous state-of-the-art approach [5].

Similarly, the results obtained with the YOLO Para models follow trends analogous to those of the off-the-shelf models while surpassing the highest-scoring YOLOv8m and YOLOv11m. In particular, the YOLO Para SP architecture reaches an AP50 of 68.8%. However, YOLO Para models are characterized by approximately four times the GFLOPs compared to the two best off-the-shelf models.

Finally, YOLO-Tryppa emerges as the best-performing model in terms of AP50, achieving a score of 71.3%, on average, with a standard deviation of 0.3 across five runs keeping the original train, test, and validation dataset splits. Remarkably, it requires only 11.3 million parameters (the lowest among the selected architectures) and 77.1 GFLOPs, despite incorporating the computationally demanding *P2* prediction head. Due to the high computational cost, we restricted our detailed evaluation to the final YOLO-Tryppa architecture, conducting five iterations using the original train, test, and validation splits provided in [5]. For this architecture, we performed a one-sample *t*-test to assess the significance of our AP50 results. All results yielded *p*-values below 0.05, with the highest being $1.86\times 10^{-5}$.

A detailed analysis based on the averaged confusion matrix over five iterations reveals that, on average, the YOLO-Tryppa model detected approximately 6426 instances of Trypanosoma parasites while missing around 2993 instances. In addition, it falsely identified about 2271 background instances as parasites. These numbers indicate that the model achieves robust performance with a high true-positive detection rate accompanied by a relatively low number of false positives, underscoring its effectiveness in parasite detection. Furthermore, our analysis of false positives and false negatives reveals important clinical implications. When background elements are incorrectly classified as parasites, patients may be subjected to unnecessary follow-up procedures and experience additional stress. Conversely, missed detections of actual parasites could delay diagnosis and treatment, potentially leading to detrimental health outcomes.

### 4.4. Ablation Study

An ablation study was conducted using the YOLOv11m baseline as a reference to evaluate the impact of individual architectural components.

Table 2 summarizes the changes in the AP50 metric resulting from our proposed key modifications. Starting from the YOLOv11m baseline AP50 of 68.4%, replacing standard convolution layers with ghost convolutions slightly reduced the AP50 to 67.2%, despite significantly lowering the parameter count and GFLOPs. In contrast, adding the dedicated *P2* prediction head, specifically designed to improve the detection of small objects, increased the AP50 to 69.2%. Further changes, such as integrating a CBAM [41] module or adding a *P1* prediction head for extremely small objects, caused a decline in performance. Consequently, the final YOLO-Tryppa architecture, which incorporates ghost convolutions and the *P2* prediction head, in addition to the removal of the *P5* prediction head without an additional CBAM or *P1* head, delivers the best balance. This configuration achieves an AP50 of 71.3%, the lowest parameter count, and reduced computational complexity. These results highlight that carefully selecting and refining architectural components can significantly enhance the detection of small Trypanosoma parasites.

### 4.5. Qualitative Results

**Figure 4:**
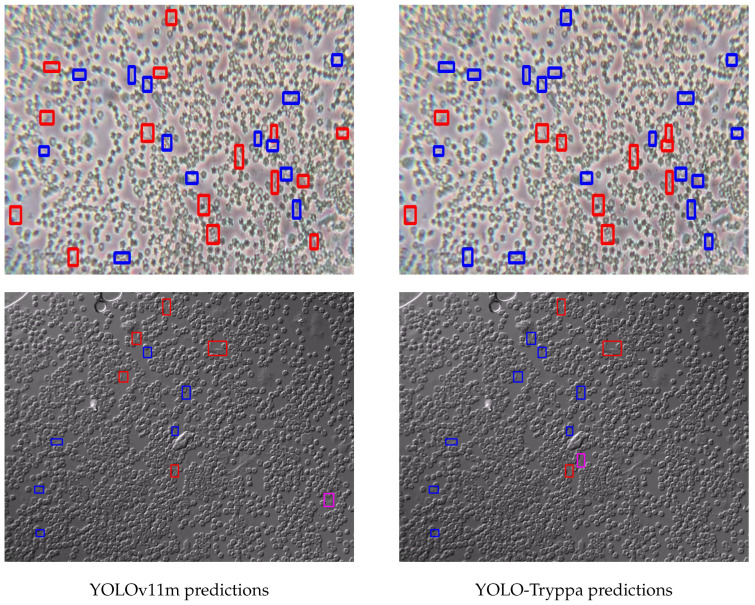
Predictions obtained from both YOLOv11m and YOLO-Tryppa. TPs are shown in blue, FPs in pink, and FNs in red.

In addition to quantitative evaluations, we present a qualitative analysis to further demonstrate the effectiveness of YOLO-Tryppa. Figure 4 presents representative examples where the model successfully localizes Trypanosoma parasites under challenging conditions, including low contrast, overlapping structures, and noisy backgrounds. These results demonstrate the model’s ability to capture subtle localized features, primarily enabled by the dedicated *P2* prediction head, which reduces FPs and ensures precise detection.

A clear trend emerges from the examples: while both models tend to under-predict, YOLO-Tryppa consistently outperforms its off-the-shelf counterparts by detecting a higher number of parasites. This improvement is visually apparent in Figure 4 and quantitatively supported by the highest recall score.

### 4.6. Discussion

The outcomes achieved with the YOLO-Tryppa framework not only highlight advancements in Trypanosoma parasite detection capabilities but also carry significant implications for theory and practice. From a theoretical standpoint, our findings contribute to the advancement of knowledge regarding the impact of architectural modifications in deep learning, particularly for enhanced detection of small objects. This insight adds depth to the field of custom convolutional neural networks for specific tasks, suggesting that intentional adjustments can lead to notable enhancements in performance. On a practical level, the effective application of YOLO-Tryppa in diagnostic settings underscores the necessity of integrating automated technologies in health care, especially in areas with limited resources. The framework’s efficiency and precision can expedite the diagnostic process, potentially resulting in improved health outcomes for individuals affected by neglected tropical diseases. Moreover, the implementation of such systems can alleviate the workload of healthcare professionals, enabling them to focus on more complex medical challenges.

## 5. Limitations

The proposed YOLO-Tryppa framework, despite demonstrating improved performance in detecting small Trypanosoma parasites, has several inherent limitations. One notable constraint is the limited diversity and scale of the Tryp dataset, which may not fully capture the broad variability encountered in real-world clinical samples. Additionally, substituting standard convolutional layers with ghost convolutions, although effective in reducing computational cost, can compromise the model’s ability to capture complex feature details in highly challenging imaging conditions. The current architecture’s performance under extreme noise, significant variations in parasite morphology, or scenarios with overlapping structures remains to be further validated. Future research should consider integrating more robust data augmentation techniques and additional architectural refinements to address these challenges and improve the overall generalizability of the model.

## 6. Conclusions

In conclusion, YOLO-Tryppa represents a significant advancement in the automated detection of Trypanosoma parasites in microscopy images. By leveraging ghost convolutions and a dedicated *P2* prediction head, the framework achieves a balanced trade-off between high detection accuracy and computational efficiency, outperforming several baseline models while maintaining a low parameter count and GFLOPs, outperforming the previous state of the art by achieving an AP50 of 71.3% compared to the previous 63.0%. The experimental results underscore the potential of this tailored deep learning approach in addressing critical challenges in medical diagnostics, particularly in resource-constrained environments. Despite the noted limitations, the promising outcomes of YOLO-Tryppa encourage future enhancements and its integration into clinical workflows, ultimately contributing to more rapid and reliable disease screening and improved patient care.

## Figures and Tables

**Table 1:** Comparative evaluation results obtained on the test set of the Tryp dataset.

Method	Image Size	Precision (%) ↑	Recall (%) ↑	F1 (%) ↑	AP50 (%) ↑	AP (%) ↑	Parameters (M) ↓	GFLOPs ↓
Baseline RetinaNet [5]	1333×800	-	-	-	50.0	-	-	-
Baseline Faster R-CNN [5]	1333×800	-	-	-	63.0	-	-	-
Baseline YOLOv7 [5]	640×640	-	-	-	55.0	-	36.9	104.7
YOLOv5m	640×640	71.6	59.3	64.9	66.0	30.7	21.2	**49.0**
YOLOv5l	640×640	70.0	57.5	63.1	64.1	30.5	46.5	109.1
YOLOv8m	640×640	72.9	62.2	67.1	68.4	32.5	25.9	79.3
YOLOv8l	640×640	61.4	49.5	54.8	54.4	24.5	43.7	165.7
YOLOv11m	640×640	71.5	63.0	67.0	68.4	31.4	20.0	68.2
YOLOv11l	640×640	72.7	61.6	66.7	68.0	31.4	25.4	87.6
YOLO Para SP	640×640	72.9	63.6	67.4	68.8	33.9	38.9	237.3
YOLO Para SMP	640×640	73.2	60.3	66.1	66.9	31.1	51.5	142.5
YOLO Para AP	640×640	69.1	60.7	64.6	66.0	32.0	66.7	161.9
YOLO-Tryppa	640×640	**73.7 ± 0.7**	**66.7 ± 0.6**	**70.0 ± 0.3**	**71.3 ± 0.3**	**35.9 ± 0.3**	**11.3**	77.1

**Table 2:** Ablation study on YOLOv11 variants. The table highlights the use of different architectural additions, where a ghost convolution corresponds to substituting convolutional layers with ghost convolution and CBAM corresponds to adding a CBAM layer before each prediction head.

Model	Ghost Convolution	P2 Prediction Head	CBAM	P5 Prediction Head Removed	P1 Prediction Head	AP50 (%) ↑	Parameters ↓	GFLOPs ↓
YOLOv11m						68.4	20.0	68.2
YOLOv11m	✓					67.2	16.7	**63.8**
YOLOv11m	✓	✓				**69.2**	14.6	79.8
YOLOv11m	✓	✓	✓	✓		66.6	14.8	80.1
YOLOv11m	✓	✓	✓	✓	✓	63.6	16.8	112.3
YOLO-Tryppa	✓	✓		✓		**71.3 ± 0.3**	**11.3**	77.1

## Data Availability

All the material used and developed for this work is available in a GitHub repository (https://github.com/unica-visual-intelligence-lab/YOLO-Tryppa, accessed on 9 April 2025).

## Abbreviations

The following abbreviations are used in this manuscript:

NTDs	Neglected tropical diseases
CNNs	Convolutional neural networks
YOLO	You Only Look Once
CAD	Computer-aided diagnostic
AP	Average precision
TP	True positive
FP	False positive
FN	False negative
P	Precision
R	Recall
